# Symbiotic relationships through longitudinal integrated clerkships in general practice

**DOI:** 10.1186/s12909-022-03119-x

**Published:** 2022-01-26

**Authors:** Andrew O’Regan, Jane O’Doherty, James Green, Sarah Hyde

**Affiliations:** 1 School of Medicine, Health Research Institute, Faculty of Education and Health Sciences University of Limerick, Limerick, Ireland; 2 School of Allied Health, Health Research Institute,, Faculty of Education and Health Sciences University of Limerick, Limerick, Ireland

**Keywords:** Longitudinal integrated clerkships, General practice, Doctor-patient relationship, Primary care, Doctor-patient relationship

## Abstract

**Background:**

Longitudinal integrated clerkships (LICs) are an innovation in medical education that are often successfully implemented in general practice contexts. The aim of this study was to explore the experiences and perspectives of general practitioner (GP)-tutors on the impact of LICs on their practices, patients and the wider community.

**Methods:**

GPs affiliated with the University of Limerick School of Medicine- LIC were invited to participate in in-depth interviews. Semi-structured interviews were conducted in person and over the phone and were based on a topic guide. The guide and approach to analysis were informed by symbiosis in medical education as a conceptual lens. Data were recorded, transcribed and analysed using an inductive thematic approach.

**Results:**

Twenty-two GPs participated. Two main themes were identified from interviews: ‘roles and relationships’ and ‘patient-centred physicians’. Five subthemes were identified which were: ‘GP-role model’, ‘community of learning’, and ‘mentorship’, ‘student doctors’ and ‘serving the community’.

**Conclusion:**

LICs have the potential to develop more patient-centred future doctors, who have a greater understanding of how medicine is practised in the community. The LIC model appears to have a positive impact on all stakeholders but their success hinges on having adequate support for GPs and resourcing for the practices.

**Supplementary Information:**

The online version contains supplementary material available at 10.1186/s12909-022-03119-x.

## Introduction

Symbiotic relationships are the catalyst for medical education to make a difference in communities. One such relationship is that between physician and student, whereby the physician practising medicine view it as their duty to train physicians of the future. General practice, with its access to huge volumes of patients, continuity of care and diversity of clinical presentations, has much to offer the physician-apprentice. In the UK, 90% of consultations in the NHS take place in general practice, but the profession is undergoing a ‘workforce crisis’ [1], with growing workloads and difficulty retaining general practitioners (GPs) [2]. Similarly, in Ireland, expert reports have recommended that more undergraduate and postgraduate training take place in general practice, partly to alleviate workforce capacity concerns [3, 4]. In this context, medical education in general practice is described as a “double-edged sword” for participating practices [5], with advantages for staff morale and practice reputation on one hand, that are offset against loss of productivity on the other [6]. There is huge variability between universities in the proportion of students opting for general practice as a career [7, 8]. Studies have identified two significant factors influencing medical students who opt for general practice as a career choice: exposure to general practice in the curriculum, and to GPs as role models [8, 9].

In this context, the School of Medicine at the University of Limerick (UL) was established in 2007; it has a strong general practice footprint, including an early-patient contact programme in year two, and an 18-week longitudinal integrated clerkship (LIC) in general practice in year 3 [10]. Traditionally, medical schools have provided short ‘block rotations’ in general practice, with limited opportunity for active participation or mentorship. The LIC model, with extended duration of placement, facilitates participation of medical students in patient care, and development of relationships with GP-tutors [11, 12]. LICs provide excellent learning environments by delivering an immersive curriculum, where students play an active role, taking on responsibility and becoming part of the practice team. Further, research conducted among graduates of the University of Limerick School of Medicine, reported that 43% of respondents chose a career in general practice [13].

The literature on LICs is expanding, with over 40 research articles published, the majority from North America and Australia [14]. The authors are not aware of literature reporting on experiences of LICs in Ireland. This deficit is notable in the context of workforce capacity problems, dwindling morale among GPs [15], and the potentially negative influence of traditional medical school informal curricula on career choice [16]. Understanding the experience of GPs will contribute to modern health planning and medical education, so that the LIC model can be improved and some of its features can be incorporated into more traditional forms of placement. The research question, therefore, is how do GPs perceive the impact of LICs that they facilitate in their practices? In the context of the importance of mutually beneficial relationships both to well-functioning general practice and apprentice-style approaches to medical education, the authors chose the theory of symbiosis as a theoretical lens for this research [17]. The aim of this study was to explore experiences of GP-tutors who provide LIC-placements and to understand the impact of the LIC model on:

1. the student, practice and GP-tutor
2. the patient and wider community

## Method

### Theoretical lens

The symbiosis model emphasises achieving mutual benefit for all stakeholders. It is used as a conceptual lens to investigate GP-tutor experiences in this study. It places student learning at the centre of relationships between personal and professional; clinician and patient; health service and medical school; government and community [17]. Different theories will illuminate different elements of a phenomenon and the symbiosis theory is useful when studying LICs as it promotes consideration of important elements of relationships between different the different stakeholders involved.

### Study design

This study is reported in accordance the principles of the Declaration of Helsinki and reporting followed the COREQ guidelines [18]. Ethical approval granted by University of Limerick Health Sciences Research Ethics Committee (EHS_2014_02_16). The study is part of a broader study investigating attitudes on research and teaching among GPs affiliated with the UL [19]. The UL LIC is a mandatory part of the curriculum and involves students being immersed in a single General Practice for an 18-week period, with one named GP supervisor per student.

The study employed an exploratory qualitative design, using in-depth, semi-structured interviews. Participants were located in three of Ireland’s four health regions, and recruited by purposive sampling, to achieve balance in years of experience in clinical practice and medical education, rurality, gender and practice size. Recruitment was via email sent by the LIC administrator and including the following: synopsis of the study, invitation to participate, email contact details for those wishing to participate or to enquire further about the study. For interview location, participants could choose between the school of medicine or their own clinics. Prior to interview, participants were sent a consent form and were asked to complete and send back to the interviewer.

### Data collection

Interview questions were based on a topic guide but designed to allow free discussion. The topic guide was devised by a team with experience in medical education, general practice, and qualitative research and further informed by a previous study of GPs’ experiences in medical education [20]. Using symbiosis, as a conceptual lens, it included questions about GPs’ experience with teaching, questions relating to the LIC model, and impact on themselves, practice staff and patients.

Individual semi-structured interviews were conducted in 2017 by a GP interviewer (AOR). Interviews were conducted in person either in GPs’ clinics or the School of Medicine. Interviews were recorded, transcribed and field notes were taken contemporaneously.

The interviewer was familiar with many participants, and as the Senior Lecturer on the LIC programme, he had formed views on the subject from personal experience. The interviewer engaged in a recognised process of reflexivity, clearly stating his role as researcher at the outset of interviews and engaging in reflection and discussion with the research team throughout in order to identify and consider potential sources of bias [21].

### Analysis

NVivo v12 was used for data management. An inductive approach to data interpretation was taken, based on the approach outlined by Braun and Clarke’s framework for thematic analysis [22]; this served as a framework to reach an agreed understanding of the meaning data in terms of the research question, so that themes could be generated. Analytical rigor was ensured by co-coding and peer debriefing. All members of the research team read the transcripts. Coding was conducted independently by two researchers who compared and standardised codes. Subsequently, the analysis team met to develop codes and group them into themes.

Data were interpreted by a process of iteration, whereby meaning and context of themes was understood from educational and clinical perspectives. Chunks of data were discussed, placed in context of the research question, participants and other data, to generate richer understanding. All analysts were senior qualitative researchers with experience in general practice and/or medical education. Data saturation was achieved by interview 16.

## Results

### Characteristics of GPs interviewed

A total of 22 GPs participated (representing >20% of the tutor network). Range of experience was 1 - 7 years. At time of interview, two participants had taken a step back from their tutor role and another had retired from teaching and practice. Interview duration ranged from 25-82 minutes.

Data analysis produced two overarching themes in relation to the research question. ‘Roles and relationships’ was the first and had three subthemes, including: ‘GP-role model’, ‘Community of learning’, and ‘Mentorship’. The second overarching theme, ‘Patient-centred physicians’, contains two subthemes, ‘student doctors’ and ‘serving the community’.

### Roles and relationships

‘Roles and relationships’ describes the formation of relationships necessary for learning, and how this is facilitated by stakeholders taking on specific roles.

### GP-role model

Close exposure to a single medical tutor over eighteen weeks provides a unique opportunity to observe professional behaviour of the tutor. GPs are aware of the ensuing responsibility, commenting how they are forced to reflect on how and why they do things, thus increasing self-awareness and potentially improving their performance.

It professionalises the doctor and practice a lot more. He or she realises they’re being watched. … in doing so, it improves their performance with patients… they become more professional (participant 19)

GPs emphasise the importance of developing their students’ understanding of patients as part of the goal of role-modelling:

You aren’t trying to turn them into the doctor you are but… trying to give them a certain amount of leadership… to show them what you think is clinically and ethically the right way to deal with patients… maybe you are exerting some kind of influence on the kind of doctor they will be later (participant 2)

how to interact with staff and how to interact with patients. To be a role model you have to be a human model as well. (participant 15)

While the longer duration of LICs facilitates more role-modelling, it is also more time consuming. One GP cited inability to balance educational and clinical commitments as the reason for opting out of his tutor role. For many, the satisfaction and sense of identity that role-modelling instils outweighs time concerns:

You come to realise that you have a fount of knowledge and experience that you didn’t realise you had. I am an asset in medical education. I am encouraged to keep the standard up. (participant 9)

This highlights the way teaching in General Practice can role model high standards.

### Community of learning

Creating an environment conducive to forming and maintaining relationships is a key factor for the successful implementation of a LIC. For this to happen, members of the practice must collaborate. Tutors described it as teamwork in action, with potential for everyone to learn. Development of students into team players takes time, oversight by GP tutors and practice teams, and motivation from students themselves.

It is about the role of collaborative teamwork and friendship, and how, with co-operation, and working together you can learn together. (participant 9)

Their work in the practice with the different professionals and the non-professional staff, the admin staff. You like to see them working very closely with them and how it all evolves. Over a period of time, you get better at seeing these things and you get more fine-tuned into all of it. (Participant 11)

Several participants had experience with more traditional (shorter duration) placements and were able to contrast the two models in this regard:

They [LIC students] became part of the practice, …. They [on shorter placements] were here today and gone tomorrow. (participant 21)

Challenges occur, and one participant reveals how his secretary groans when students arrive as his own timekeeping invariably deteriorates. Participants agreed that some students struggle, and they tend to be those with poorer communication and interpersonal skills, as they invariably find it harder to integrate with the practice team. With appropriate steering, most students do integrate over the duration of placement.

### Mentorship

A close relationship develops between students and GP-tutors, with GPs acting as a source of support and information, often extending beyond LICs. There was a sense that the GP-tutor’s role was to develop the student. This represented a continuum of medical education that GPs themselves had benefitted from during their own training.

Some of them get on better with some of us than others. Some I am still friends with. I still have their numbers on my phone, I have written them references. If they have a big hankering for, let’s say, paediatrics I have introduced them to friends of mine who do it. It’s not just the relationship with the GP, it’s the whole team. (participant 13)

This quote illustrates how mentorship ties into the subtheme of community of practice and shows how GP-tutors see themselves as medical educators. The process of supporting students as they grow in confidence across a range of competencies is described:

Gradually...you can see them flowering, up-skilling, developing confidence in themselves as time goes on, so that by the end of year three they have a broad set of competencies in… general practice, and medicine and surgery for that matter. (participant 9)

Mentoring relationships — being dependent on personalities among other complex factors — are sometimes unsuccessful and many participants expressed need for support from the university and guidance on managing problematic relationships. Despite that, negative experiences with students were rare and were not a factor in GPs ceasing to participate as tutors.

### Patient-centred physicians

‘Patient-centred physicians’, analyses how learning on LICs can contribute to forming doctors who are patient focussed.

### Student-doctors

Participants describe students developing from passive learners to active participants who help with practice workload. Not all had this experience, some tutors described the draining impact on their time throughout the 18-weeks. There was consensus that — for students who were interested — development in their role did occur, but it didn’t always impact on practice workload. The important point for GP-tutors was that students develop relationships with patients, to understand patients’ stories and to begin to advocate for them. The term ‘student doctor’ seemed appropriate to describe this.

They can contribute to the care of the patient, maybe not as much as a qualified doctor or nurse but they still contribute by listening, examining, note checking, file checking, doing some background research on conditions … so speed up work in the practice. (participant 20)

Participants were aware of benefits to students as doctors of the future:

If they are tuned into patients, tuned into themselves, tuned into the job, they aren’t just going through the motions. They are better doctors for it. They are less likely to get burned out. They are not machines on the job. (participant 13)

There was agreement that patients were generally receptive to students, getting to know them and sometimes revealing more detailed histories to them.

### Serving the community

The benefit to students and individual patients extends beyond consulting rooms and out to communities, with wider societal benefits.

Students learn to understand communities. How rural our community is, how people enjoy it and what makes the people tick. That’s important (participant 11)

Another participant described how the LIC facilitated an understanding of medicine that shorter placements didn’t:

It’s something we could never do with students that we had for one or two weeks because we couldn’t try to explain to them what was happening with the long term follow up of patients… to get them to see the social side of things and how that impacts on healthcare… to learn what are the challenges that might be faced by patients and health care staff in the community (participant 8)

GPs, through role-modelling and mentorship, aid development of doctors with higher levels of community awareness. Some participants felt that their contribution to the formation of tomorrow’s doctors is not understood or appreciated. For it to be sustainable, general practice should be resourced and supported.

you can't cut money to Primary Care by 40% and continue to turn out top graduates (participant 19)

## Discussion

### Summary

Data analysis addressed the research questions through the lens of symbiosis and shows that LICs depend on relationships forming between stakeholders, with each playing specific roles. Stakeholders include GP-tutors, practice staff, patients and wider society. This is mutually beneficial for those involved but there are exceptions, and the benefit comes at cost to GPs in terms of time. The data demonstrate the powerful influence GP-tutors can exert on the formation of future doctors, and there was a sense that this is underappreciated. If GP tutors are adequately supported and resourced, they can facilitate LICs that shape future doctors in a very positive way, and their relationships with patients.

### Comparison to current literature

To achieve symbiosis, barriers to teaching need to be identified. Research has reported barriers to teaching in general practice, including time pressure on practices and patient fatigue in engaging with students [23]. Our data suggests that over the duration of each student’s clerkship the time demand dissipates in most but not all instances. Our data outlines how practice teams become more cohesive in response to LICs, facilitating medical students to be members of the community of practice, moving from peripheral observers to central members. The socialisation process into a community of practice is facilitated by GP tutors and other team members.

Relationships between students, supervisors and patients are at the centre of LIC programmes, and several models for this triangular relationship have been described [24–26]. GP-tutors in this study found that when students are given a safe space and opportunity to build relationships with patients, it can result in patients returning to students to discuss their medical concerns. Research reporting patients’ experiences with students on LICs has been positive, with patients appreciating the attitudes of students and perceive their overall care to be enhanced [27]. It is clear from the literature that appropriate support, supervision and opportunity for both students and tutors to reflect on their experience are needed to optimise relationships within LICs [28, 29]. Reflection, as described by Fish and De Cossart, can be approached in a systematic way, as a catalyst for professional growth for doctors [30].

Prideaux’s concept of symbiosis as a model for education incorporates many of the concepts that this study has reported [17]. The idea of mutually positive relationships between stakeholders providing excellent learning opportunities in real-life settings comes across strongly in our data. Our participants describe satisfaction, improvement in morale and greater sense of professionalism. Tutors in more traditional models may also report these benefits but our data points very clearly that they are enhanced by the LIC experience. This may be because time is needed, to develop relationships, and for GPs to feel like ‘co-learners’ in the process.

Using the theory of symbiosis, with its emphasis on bi-directional relationships at several levels, has facilitated a practical and in depth understanding of LICs from the GP-tutor perspective. Advocates of LICs have described symbiosis as both a guiding principle for medical education [31] and as an enabler of professional identity formation [32]. Symbiosis overlaps with other theories that emphasise the centrality of relationships that have been applied to the LIC model. Communities of practice is a theory that views learning in longitudinal integrated clerkships as a process of socialisation [33]; in this instance the community of practice refers to as a “group of people who share a concern for… something they do and learn how to do it better as they interact regularly” [34]. This relates to the subtheme in our study, ‘communities of learning’ is illustrated by the quote “*with co-operation, and working together you can learn together”.*

A particular type of relationship which appeared as a theme in our data analysis is mentorship. The mentor-mentee relationship in medical education was the subject of a review by Frei et al, who defined mentorship in this context as: “a relational process in which five phases can be distinguished: information on career options, developing career plans, focusing on career goals, realization of career steps, and evaluation of career advancement” [35]. The mentoring relationship is differentiated from other types of relationships, such as tutoring, role-modelling and coaching in that it is “supportive and often protective” [35].

It is important that the mentoring, role-modelling and education provided by GPs is valued. The reference, from one participant, to cutting the primary care budget by 40% and expecting GPs to “continue to turn out top graduates” suggests that for this model to be sustainable, recognition, resourcing and support from stakeholders in health and medical education are necessary. The literature reports that GPs are motivated to help the next generation of GPs and attract doctors to their community [36].

### Implications for research and practice

Many medical schools will not be in a position to implement LICs, but there are lessons that can be considered: practice team approach to integrating students; patient-centred approach to learning; incorporating factors that affect health of communities into curricula; emphasis on reflection, greater awareness of attitudes and how they affect development. LICs may increase recruitment to general practice and may improve understanding of general practice among hospital colleagues who engage. Research is needed to investigate if this is happening and to explore how it develops beyond LICs into postgraduate training. We have identified instances where LICs are challenging and where tutors leave their role. Future research is needed to enhance understanding of why this occurs.

### Strengths and limitations

The large number of participants and inclusion of GP-tutors who had stepped back from teaching gives a wide and nuanced perspective. Limitations include there being only one LIC in the jurisdiction and the structure of this LIC and the health system may be very different from those elsewhere. As such, the generalisability of all of the findings is limited, but the authors maintain that the relationship factors illuminated by this analysis are very likely to be applicable to other environments. The interviewer was familiar with participants, but potential for bias was reduced by attention to reflexivity and involving researchers with experience in education and from backgrounds other than general practice and medicine. Research has reported that experts are more likely to divulge detailed experiences to interviewers they know have strong understanding [37].

## Conclusions

The two overall themes identified, ‘roles and relationships’ and ‘patient-centred physicians’, illustrate how LICs can develop more patient-centred future doctors, who understand how medicine is practised in communities. The LIC model appears to positively impact all stakeholders. Success will hinge on providing support for GPs and resourcing practices to build the future workforce.

## Supplementary Information

**Additional file 1.** **COREQ Guidelines**.

## Data Availability

All relevant data is published in the paper as illustrated quotes. For inquiries regarding data supporting the findings presented in the manuscript, please contact the corresponding author.

## Abbreviations

LIC: Longitudinal integrated clerkship
GP: General practitioner
